# IMGT/mAb-KG: the knowledge graph for therapeutic monoclonal antibodies

**DOI:** 10.3389/fimmu.2024.1393839

**Published:** 2024-06-20

**Authors:** Gaoussou Sanou, Taciana Manso, Konstantin Todorov, Véronique Giudicelli, Patrice Duroux, Sofia Kossida

**Affiliations:** ^1^ The International ImMunoGeneTics Information System (IMGT), National Center for Scientific Research (CNRS), Institute of Human Genetics (IGH), University of Montpellier (UM), Montpellier, France; ^2^ Montpellier Laboratory for Computer Science, Robotics and Microelectronics (LIRMM), University of Montpellier, National Center for Scientific Research (CNRS), Montpellier, France

**Keywords:** immunogenetics, immunoinformatics, therapeutic monoclonal antibody, ontology, knowledge graph, semantic web, visualization

## Abstract

**Introduction:**

Therapeutic monoclonal antibodies (mAbs) have demonstrated promising outcomes in diverse clinical indications, including but not limited to graft rejection, cancer, and autoimmune diseases lately.Recognizing the crucial need for the scientific community to quickly and easily access dependable information on monoclonal antibodies (mAbs), IMGT®, the international ImMunoGeneTics information system®, provides a unique and invaluable resource: IMGT/mAb-DB, a comprehensive database of therapeutic mAbs, accessible via a user-friendly web interface. However, this approach restricts more sophisticated queries and segregates information from other databases.

**Methods:**

To connect IMGT/mAb-DB with the rest of the IMGT databases, we created IMGT/mAb-KG, a knowledge graph for therapeutic monoclonal antibodies connected to IMGT structures and genomics databases. IMGT/mAb-KG is developed using the most effective methodologies and standards of semantic web and acquires data from IMGT/mAb-DB. Concerning interoperability, IMGT/mAb-KG reuses terms from biomedical resources and is connected to related resources.

**Results and discussion:**

In February 2024, IMGT/mAb-KG, encompassing a total of 139,629 triplets, provides access to 1,489 mAbs, approximately 500 targets, and over 500 clinical indications. It offers detailed insights into the mechanisms of action of mAbs, their construction, and their various products and associated studies. Linked to other resources such as Thera-SAbDab (Therapeutic Structural Antibody Database), PharmGKB (a comprehensive resource curating knowledge on the impact of genetic variation on drug response), PubMed, and HGNC (HUGO Gene Nomenclature Committee), IMGT/mAb-KG is an essential resource for mAb development. A user-friendly web interface facilitates the exploration and analyse of the content of IMGT/mAb-KG.

## 1 Introduction

To effectively combat biological attacks caused by infectious agents like viruses, bacteria and self-corrupted or infected cells, our body generates antibodies. These antibodies identify and initiate the process of eliminating these infectious agents. To replicate the natural function of antibodies, scientists created synthetic versions known as monoclonal antibodies (mAbs). They have shown interesting results for different therapeutic goals including graft rejection, the treatment of autoimmune and infectious diseases, cancers, and inflammation (1, 2). For example, during the worldwide epidemic of the respiratory disease COVID-19, in addition to the vaccine, different mAbs were proposed to prevent, treat, or avoid the severe form of the disease (3, 4). To date, 172 mAbs are used in clinical indications, and more than a thousand are under development (5). Demonstrating vast medical capabilities, the mAbs development represents a challenge and a hopeful horizon in the field of medical therapies.

To address these challenges, it is necessary to reconcile the development of mAbs with the organization and accessibility of knowledge related to mAbs. Thus, IMGT®, the international information system in immunogenetics and immunoinformatics proposed IMGT/mAb-DB: a unique expertise resource on mAbs with diagnostic or therapeutic indications (5). IMGT/mAb-DB is manually curated and is consistently updated with the World Health Organization-International Nonproprietary Names (WHO-INN) list of pharmaceutical substances or active pharmaceutical ingredients (1). IMGT/mAb-DB provides access to details about the mAbs including names, receptor type, target of the mAb, clinical indication of the mAb, among others. Users can access this information through a form-like web interface. While the user-friendly nature of this access can be advantageous, it may also impose limitations on querying capabilities and could lead to the isolation of IMGT/mAb-DB’s information from other IMGT databases. In fact, form-like queries impose to use only the queries available in the interface, while the user may need to make new queries independently on the form. For example, a user may need to query *a specific mAb, its structural information from IMGT’s databases and the genomics information like genes and alleles from IMGT/GENE-DB*. The user may need to understand and compare the information inside the database like statistics about mAbs such as *the proportion of different specificity classes, the proportion of different clinical phases in clinical trials, etc.* Finally, the user may need to see the interaction between different mAbs such as the common targets, diseases, receptors etc.

Knowledge graphs (KG) are data graph representations describing real-world entities and their interrelations (6). They allow the integration and the federation of different data sources, thus unifying their content. Recently, they have emerged as one of the most effective and efficient methods for data or knowledge integration, and have gained widespread acceptance in both academic and industry circles (7). For example, last year we created IMGT-KG, a KG aiming to unify knowledge in immunogenetics (8) by integrating immunogenetics protein structures and genomics data. We propose IMGT/mAb-KG, a KG for monoclonal antibodies integrating data from IMGT/mAb-DB and the IMGT genomics and protein structure databases. In this way, IMGT/mAb-KG is naturally connected to IMGT-KG (8), the first initiative of connecting IMGT databases in a KG. Thus, the user will be able to query mAbs information such as structure and link to the related genomics information including gene, allele, nucleotide sequences.

As with IMGT-KG’s development, IMGT/mAb-KG is built and published following the W3C recommendations (9). It reuses the IMGT-KG data model (IMGT-ONTOLOGY) and imports terms from various biomedical resources for interoperability purposes. The data are extracted and collected from IMGT/mAb-DB and some related external sources such as PharmGKB to feed the data model. The generated KG consistency is then checked with the reasoner (tool to check knowledge or to deduce new knowledge in a KG). In addition, we introduced a new interface for visualizing the IMGT/mAb-KG content center to main entities such as the mAbs, the targets, the clinical indication and the product of the mAbs. In this way, the user can not only navigate between the different entities, but can also see their interactions. Furthermore, this interface allows the user to check the statistics about different entities in the graph such as clinical phase, receptors used, etc. Currently, IMGT/mAb-KG provides access to 1,867 concepts or classes and 114 properties linking 21,842 entities.

The rest of the paper is structured as follows. In Section 1, we present our method to construct IMGT/mAbKG by providing, firstly in the Subsection 1.1, a background about antibodies in general and monoclonal antibodies in particular. Then we detail our construction method in the Subsection 1.2: from IMGT/mAb-DB to IMGT/mAb-KG. The Section 2 presents the results by providing some statistics about IMGT/mAb-KG in Subsection 2.1 while the Subsection 2.2 gives an overview of the use-cases of the graph. Finally, we discuss these results in Section 3 and give future work directions.

## 2 Methods

### 2.1 Background: adaptive immune system and therapeutic monoclonal antibodies

The immune system comprises a diverse array of cells, molecules, and mechanisms that activate in defense against external threats, such as bacteria, fungi, parasites, viruses, toxins and cancer cells, identified by foreign antigens (markers perceived as detrimental to our body) (10). This system operates primarily through two main mechanisms: innate and adaptive immunity.

The innate response is the body’s primary line of defense. This general, non-specific response is antigenindependent, involving physical barriers such as the skin and mucous membranes, as well as physiological deterrents like body temperature and pH levels. Processes such as endocytosis, phagocytosis, and the inflammatory mechanism are integral to this response. Importantly, innate immunity quickly mobilizes various immune cells to infection sites. These include phagocytes, dendritic cells, neutrophils, mast cells, basophils, eosinophils, natural killer cells (NK), and innate lymphoid cells (10). The adaptive immune response is complex and tailored, relying on antigen recognition. It is characterized by a remarkable immune specificity and memory, which are the properties of the B and T cells, as well as an extreme diversity of their specific antigen receptors (11). Its main functions encompass (12, 13): discerning foreign antigens from the body’s own, orchestrating specific immune reactions to identify pathogens, and promoting an immunological memory. This memory enhances the efficiency of the system, ensuring a faster and more effective reaction during subsequent encounters with the same pathogen. Central to the adaptive immune response are T and B lymphocytes and their corresponding receptors: the T-cell receptors (TR) for T lymphocytes and immunoglobulins (IG, also known as an antibody) for B lymphocytes.

An antibody is a heterodimer made up of four polypeptides: two identical heavy (H) chains and two identical light (L) chains. The heavy chain contains a variable heavy domain (VH) at the N-terminal end and three or four constant domains (CH1 to CH4) at the C-terminal (11). The light chain also consists of a variable light domain (VL) and a constant domain (CL). The VH-CH1 of the heavy chain associated with the VL-CL of the light chain constitutes the antigen binding fragment (Fab) involved in the recognition and binding of antigens (11). The variable domain of the Fab is made of framework regions (FR) and complementarity determining regions (CDR): hypervariable regions which are domains or loops on immunoglobulin in direct contact with antigen and frequently mutated to allow diverse antigenic specificities to be recognized. The rest of the C-terminal domains of the heavy chain form the fragment crystallizable (Fc) region that interacts with effector molecules such as the complement component C1q, leading to the elimination process of antigen (coated with antibodies). The complement is a series of plasma proteins that, when activated, contribute to the destruction of bacteria, the removal of immune complexes of antigen-antibody, and the release of molecules that attract immune cells.

The mAbs are derived from a single plasma cell. Unlike polyclonal antibodies originating from multiple plasma cells: activated B lymphocytes, mAbs are synthesized artificially in laboratories and are specific to a single epitope on a target pathogen, making them suitable for targeted biotherapies (14, 15).

However, artificial mAbs, specially isolated from non-human species, can show high immunogenicity because they are recognized and destroyed as foreign. To minimize their immunogenicity, several types of mAb have been developed (14, 16, 17) including:

- Chimeric mAb: this type involves isolating the coding DNA for the variable regions of the heavy and light chains (VH and VL) from a mouse or rat antibody and then fusing it to the constant regions (H and L) of a human antibody.
- Humanized mAb: Here, the hypervariable regions of a mouse or rat mAb are grafted onto the VH and VL domains of the human antibody.
- Fully human mAb: This approach involves cloning the variable regions (VH and VL) of a human antibody using a vector or phage, resulting in a fully human antibody.

The advent of the mAbs represents a pivotal breakthrough in medical science: therapeutic mAbs. These antibodies have played a crucial role in the formulation of treatments for a spectrum of complex diseases such as cancers or autoimmune diseases. In fact, the mAbs have been prominently developed in cancer immunotherapy over the past several decades, demonstrating vast capabilities and presenting a hopeful horizon in the field of medical therapies (1, 18, 19): feladilimab (solid cancers), avelumab (renal cell carcinoma), relatlimab (melanoma metastatic) etc. The mAbs can operate through various mechanisms (1, 17, 19) such as blocking and agonist actions.

The blocking action consists of targeting specific receptors (involved in critical signaling pathways) either on the surface cell such as cancer or immune cells, thus inhibiting the interaction of the receptor with the natural ligand. This blocking can lead to inhibition of cell growth, induction of apoptosis (programmed cell death), or reduction in cancer cell proliferation. It can also modulate the immune response by targeting immune checkpoints (1). On the other hand, agonist action consists in binding to specific receptors and simulating the action of the natural ligand by activating the receptor, then initiating the signal transduction pathway. This action can enhance the immune response against cancer cells by leading to the proliferation of T-cells or other immune cells (1).

Beyond these primary actions, therapeutic mAbs can be harnessed to deliver drugs or radiation directly to compromised cells, improving treatment precision (14, 17). In addition, they can play a crucial role in helping the immune system identify and eliminate malfunctioning or cancerous cells. In the next section, we will present IMGT/mAb-DB the database containing mAbs and the construction of IMGT/mAb-KG, the KG of mAbs.

### 2.2 IMGT-mAb-KG construction: from IMGT/mAb-DB to IMGT/mAb-KG

Acknowledging the crucial functions and importance of the genetic aspects of the adaptive immune response and its components, IMGT has established a specialized international platform dedicated to handling the diversity and complexity of immunogenetics entities (20, 21). Over the years, IMGT has curated and provided expert analysis on data related to receptors involved in the adaptive immune response. This spans from genomic insights (IMGT/GENE-DB and IMGT/LIGM-DB) (22, 23) to protein structures (IMGT/3Dstructure-DB and IMGT/2Dstructure-DB) (24) including data on engineered therapeutic proteins or mAbs (IMGT/mAb-DB) (5, 25). To ensure the integrity and standardization of this information, the IMGT-ONTOLOGY (26) has been developed, offering a standardized vocabulary tailored to elucidate immunogenetics entities. Recently, a KG has been developed to encompass data from some IMGT databases (8). It is worth noting that IMGT/GENE-DB, a part of IMGT/LIGM-DB connected to IMGT/GENE-DB, IMGT/3Dstructure-DB and IMGT/2Dstructure-DB have already been integrated into IMGT-KG (8). The database we are focusing on here is IMGT/mAb-DB, mainly dedicated to engineered mAbs with therapeutic applications. Just as the IMGT protein structures are linked to genomics information, the mAbs inside IMGT/mAb-DB possess links to protein structures access number in IMGT/3Dstructure-DB and IMGT/2Dstructure-DB. Then, turning IMGT/mAb-DB into a KG allows the connection with the subsequent integrated information in IMGT-KG.

**Figure 1:**
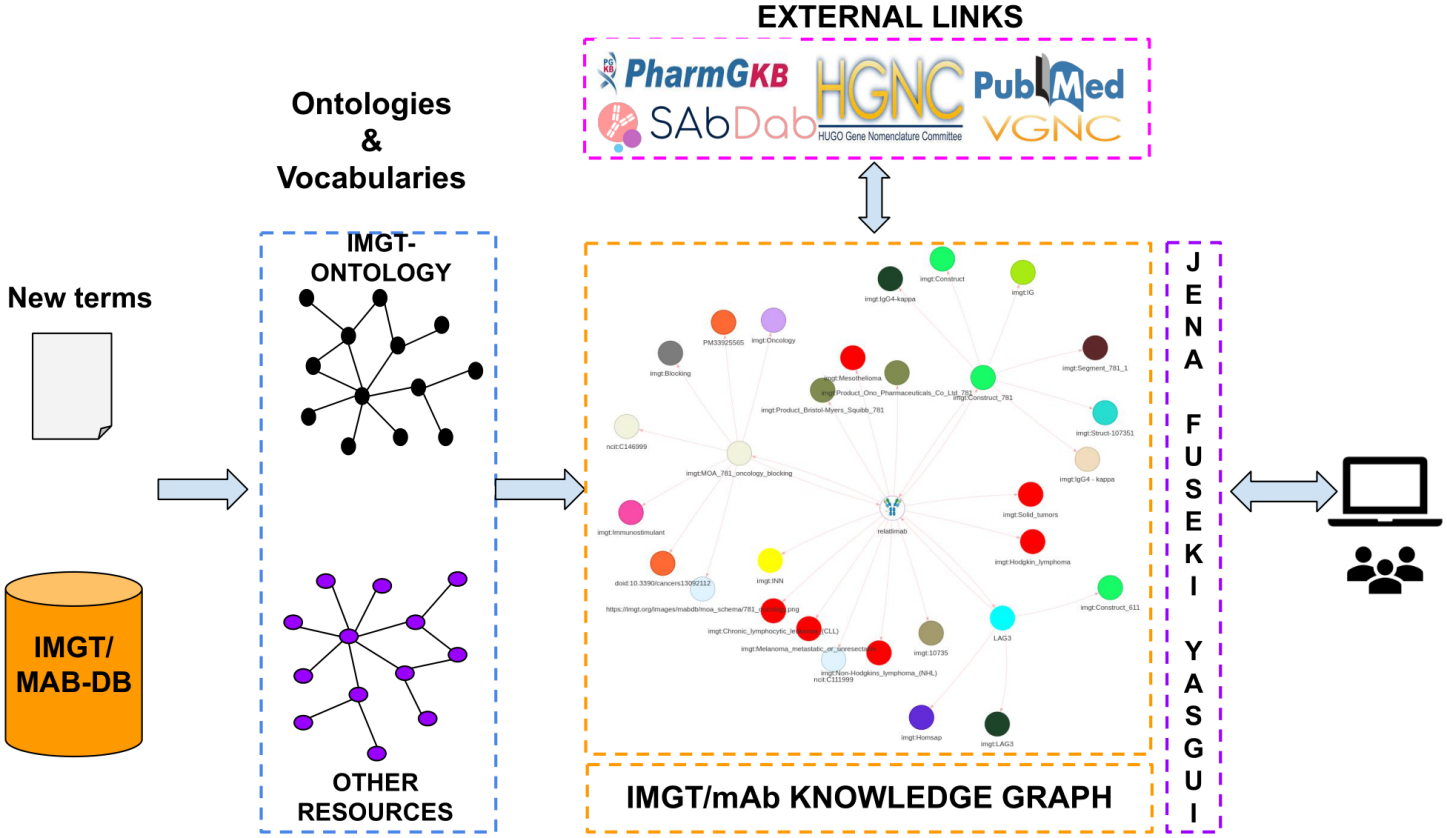
Pipeline of IMGT/mAb-KG construction.

We adopt the same construction methods as those used in (8). Briefly, we reused and extended the IMGT-KG data model based on IMGT-ONTOLOGY with new terms needed from domain resources dedicated to mAb characterization, and the data source used is IMGT/mAb-DB. The construction comprises three steps, as illustrated in **Figure 1**: i) We extend the actual IMGT-KG data model by adding new terms, ii) Then we populate the data model by adding facts from IMGT/mAb-DB, we link to external resources and generate the KG, iii) Finally, we check the consistency of the KG with the help of a reasoning engine and deduce inferred facts from the KG.

As for the development of the IMGT-KG (8), IMGT/mAb-KG has been constructed according to the W3C best practices and standards including Resource Data Framework (RDF), Resource Data Framework (RDF Schema), and Web Ontology Language (OWL). The implementation takes advantage of the Apache Jena framework for this purpose. We use OntoFox to leverage existing terminologies, an online tool that facilitates the extraction of terms from ontologies while preserving associated properties, annotations, and classes (27). The interaction with IMGT/mAb-DB is facilitated by the Object-Relational Mapping (ORM) model mapping IMGT/mAb-DB to an object model. Data access is then facilitated through the Java Persistence API (JPA). To ensure the integrity of the KG and to generate inference or new knowledge, we use the reasoner Pellet (28).

#### 2.2.1 IMGT/mAb-DB

IMGT/mAb-DB is an expertized and specialized database developed for therapeutic mAbs, fusion proteins for immune applications (FPIA), composite proteins for clinical applications (CPCA), and other related proteins of therapeutic significance (RPI) (20, 21). Acting as a gateway to therapeutic proteins detailed in the databases IMGT/2Dstructure-DB and IMGT/3Dstructure-DB, IMGT/mAb-DB is based on meticulous manual curation. Furthermore, it is consistently updated in alignment with the WHO-INN list, which provides international nonproprietary names (INN) to identify pharmaceutical substances or active pharmaceutical ingredients. As of December 2023, the database contains 1489 entries, covering 1,293 IG, 9 TR, 50 FPIA, 70 CPCA and 67 RPI.

Through its Web interface, IMGT/mAb-DB provides comprehensive insight into mAbs. This includes details from the INN, such as the proprietary name, common name, and more. In addition, it specifies the receptor type (IG, TR, FPIA, CPCA, or RPI), the origin species, the specificity of the target, clinical indications, current clinical status, a related bibliography and more recently, the mechanism of action as well as the partners and the outcomes (1). Complete information on IMGT/mAb-DB can be found at https://www.imgt.org/mAb-DB/doc.

#### 2.2.2 IMGT/mAb-KG model

In our recent study (8), we extend IMGT-ONTOLOGY (26) by integrating new terms from various domain-specific biomedical terminologies and ontologies, using them as our primary data model. The IMGT/mAb-KG data model is an extension of the IMGT-KG model, enriched with additional terms and relationships. Specifically, we incorporate terms from:

- The National Cancer Institute Thesaurus (NCIt): A comprehensive terminology source and foundational biomedical ontology encompassing various biomedical concepts, including pharmacological substances, cancer, products.
- The Mondo Disease Ontology (MONDO): An initiative focused on consolidating disease definitions on a global scale (29).
- The Bibliographic Ontology (BIBO): An ontology dedicated to the citation and bibliographic referencing realm.
- The Ontology of Clinical Research (OCRE): Curated to facilitate systematic categorization and seamless interoperability among human studies and their individual elements (30).
- The Semantic Science Integrated Ontology (SIO): an ontology that aims to facilitate knowledge discovery through simple but effective ontology-based data integration. It offers classes and relations to describe and relate objects, processes, and their attributes with specific extensions in the biomedical domain (31).
- Wikidata: a knowledge base that serves as the central repository of structured data for Wikipedia and other Wikisources.

**Figure 2:**
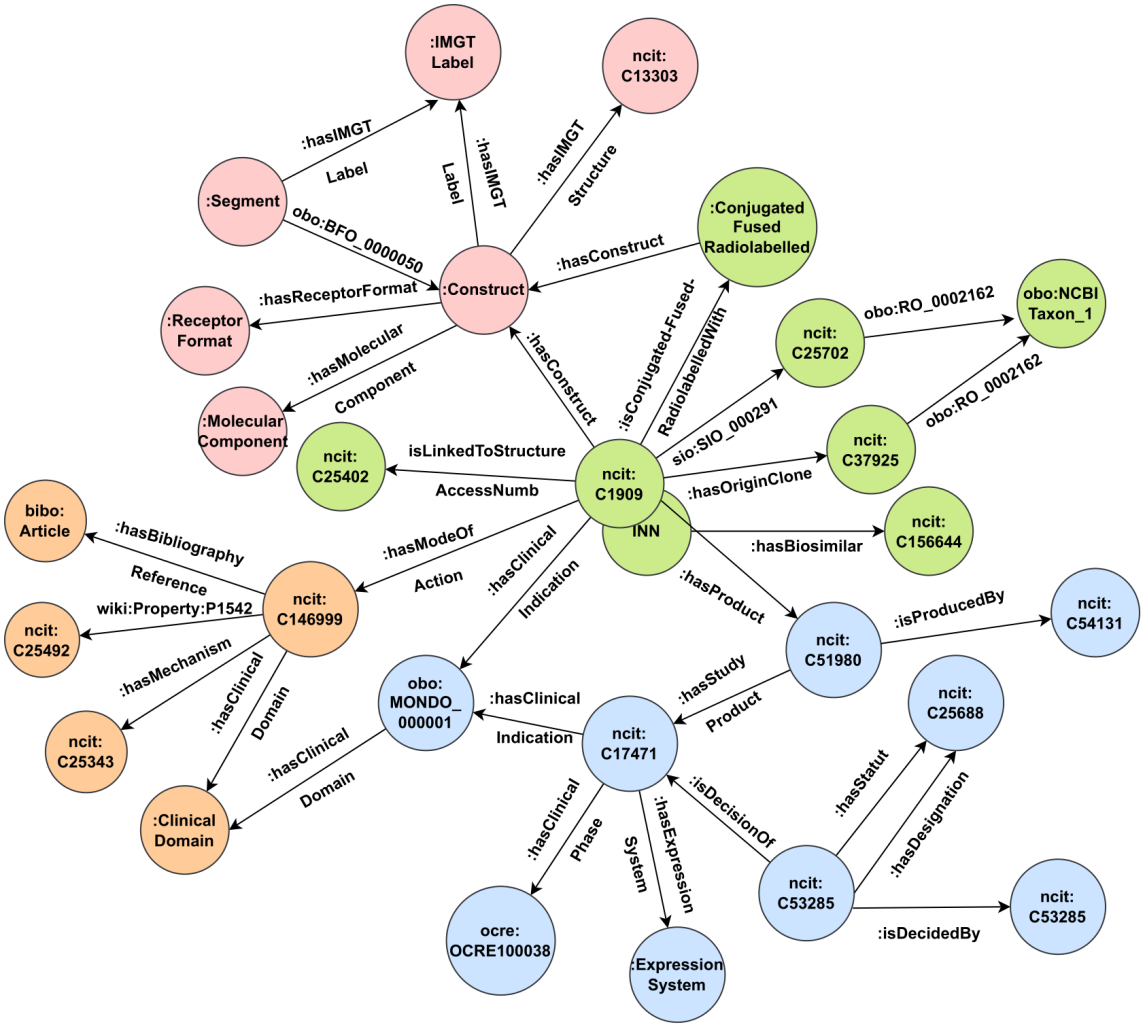
IMGT/mAb-KG data model without annotation properties. To ensure clarity, we indicate the source name (namespace) before the term when it is imported from an external source. For instance, the term ncit:C17471 refers to the term with the identifier C17471 (product) from the National Cancer Institute Thesaurus (NCIt). However, when it is an internal term from IMGT-ONTOLOGY, we do not include the namespace.

**Figure 2** describes the data model centered solely on concepts and object properties. Different levels of knowledge can be highlighted:

- Central to the data model (represented in green) are the concepts of Pharmacological Substance (ncit:C1909) and the INN, along with their related knowledge. These serve as the primary entry points for a mAb. Then a Pharmacological Substance and/or INN concept can have an origin cellular clone (ncit:C37925) from a given species (obo:NCBITaxon_1). A pharmacological substance targets (sio:SIO_0000291) a gene product (ncit:C25702) that is inherent to a species. Furthermore, a pharmacological substance can have biosimilar products (ncit:C156644). A mAb can be linked to an access number (ncit:C25402), establishing a link between IMGT-KG and IMGT/mAb-KG. A mAb could be conjugated, radiolabelled, or fused with other molecules.
- Presented in pink are the concepts of the construct and its associations. A construct refers to the composite of various elements used to compose the mAb. It can have a receptor format and a molecular component. Every construct possesses an IMGT label and at least one segment with its own IMGT label; in reverse, the segment is a part of (obo:BFO_0000050) a construct. Furthermore, a construct might be associated with a protein structure (ncit:C13303), which mirrors the protein structure used in IMGT-KG, thus bridging the two KG. In addition, we include related knowledge about protein structure (protein chain, protein domain, region, sequence alignment, allele, and other) according to the IMGT-KG data model, as explained in more detail in (8).
- The illustration in light blue captures the essence of the concept of the product (ncit:C51980) of the mAb and the knowledge it encompasses. A product is the result of a company’s production (ncit:C54131) and invariably undergoes at least one evaluation or study (ncit:C17471). Such product evaluations are undertaken for specific clinical indications, predominantly diseases (obo:MONDO_000001). The status of a product’s development is communicated through its clinical phase (ocre:OCRE1000038), and it could be expressed within an expression system. Each study product could be subject to a decision (ncit:C53285) that results in a specific designation or status (ncit:C25688) taken by an organization (ncit:C53285).
- Portrayed in orange are elements that encapsulate the concept of the mechanism of action (ncit:C146999). The mechanism of action (MOA) of a mAb encompasses a clinical domain, a defined mechanism (ncit:C25343), one or more effects (ncit:C25492), and is supported by bibliographic citations (bibo:article). The relationship between the MOA concept and its effects is defined by the has effect (wiki:Property:P1542) relation.

For clarity, we have omitted information about annotation properties in our description; this includes additional information on the mAb (INN name, INN list, clinical trials, and PharmGKB links), the product (common name, commercial name), and the study product (application type).

#### 2.2.3 IMGT/mAb-KG data model’s population

**Figure 3:**
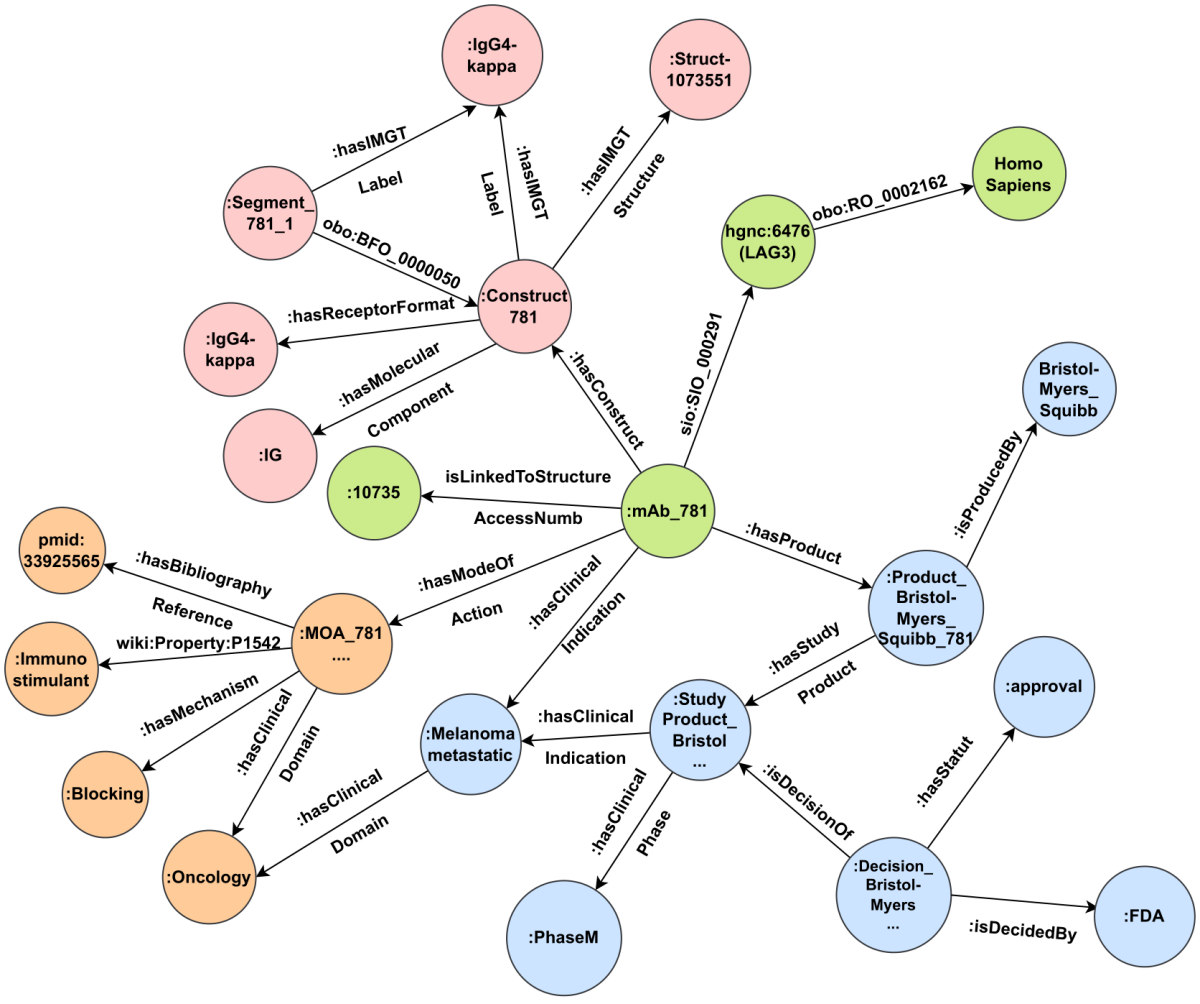
An example of the description of the mAb 781 (Relatlimab) in IMGT/mAb-KG.

Once the IMGT/mAb-KG data model is defined, we add facts to the graph using the IMGT/mAb-DB data. Then we connect to IMGT/mAb-DB and query all the mAb identifiers and their related properties. These results are used to instantiate the data model of our KG. **Figure 3** shows an example of the description of mAb 781 in IMGT/mAb-KG. mAb_781 refers to Relatlimab, is linked to the access number 10735, targets the LAG3 lymphocyte activating 3 (hgnc:6476) belonging to the *Homo sapiens* taxon. The mAb781 has the construct construct_781 and mode of action MOA_781_oncology_blocking and one of its products is the ProductBristol-MyersSquibb781.

The construct construct_781 has an IMGT label IgG4-kappa, which is also a receptor format. It is made up of one single segment, which is labeled IgG4-kappa. The construct has an IG as a molecular component and is related to the protein structure Struct-1073551, an entry of IMGT/2Dstructure-DB.

The product Product_Bristol-Myers_Squibb781 has many study products, here we have the study product: StudyProduct_Bristol-Myers_Squibb_Melanoma_metastatic_or_unresectable_781 in a phase M (market phase) used to treat the melanoma metastatic, a malignant melanoma. The Food and Drug Administration (FDA) has taken a decision to approve this study product.

The mode of action MOA_781_oncology_blocking has a blocking mechanism and has an immunostimulant effect. This mode of action is described in the article pmid:33925565 and the clinical domain is oncology.

Once the KG is generated, the next step is to check the consistency of the KG. In fact, it is important to ensure that our facts (instances) in the KG are consistent with the data model; thus, we ensure that no violation of the data model is encountered in the KG with Pellet and compute inferences to deduce new facts.

#### 2.2.4 Linking IMGT/mAb-KG to external sources

IMGT/mAb-KG is linked to some external sources via different annotation properties link, such as:

- Thera-SAbDab is a therapeutic structural antibody database containing immunotherapeutic variable domain sequences and their related structural information (32). This database hosts metadata about therapeutic antibodies. We link this resource with the mAb using therapeutic_sabdab_link, then the user can find the related information on a specific mAb in Thera-SabDAb when it exists.
- PharmGKB is a comprehensive pharmacogenomics knowledge repository, this resource consolidates critical clinical data such as drug labels and clinical guidelines. Furthermore, it includes information on gene-drug interactions that may have clinical significance, as well as relationships between genotypes and phenotypes (33). Linking is performed at the mAb and target level using annotation properties such as pharmgkb_id, pharmgkb_id_link, pharmgkb_prescribingInfo etc.
- PUBMED is a database containing biomedical literature from MEDLINE, life science journals, and online books. We used direct doi, pmc or pubmed link in the bibliography references.
- Hugo Gene Nomenclature Committed(HGNC): this international organization is responsible for approving unique symbols and names for human loci, including protein coding genes, ncRNA genes and pseudogenes, to allow unambiguous scientific communication. We use it when there exists the HUGO nomenclature link for the mAb’s targets.
- Vertebrate Gene Nomenclature Committee (VGNC) is a resource for the approved gene nomenclature of other vertebrate species.

## 3 Results

### 3.1 Statistics on IMGT/mAb-KG

**Figure 4:**
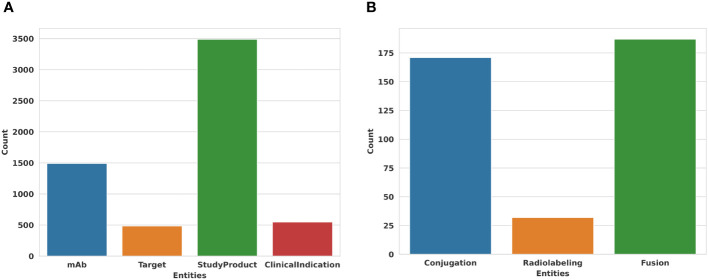
Some statistics of entities inside IMGT/mAb-KG. **(A)** Main entities in IMGT/mAb-KG. **(B)** Specific modifications in mAb inside IMGT/mAb-KG.

With 139,629 triplets, IMGT/mAb-KG counts 1,867 concepts or classes and 114 properties or relations used to link 21,842 entities. IMGT/mAb-KG counts approximately 35,000 study products for around 1,500 mAbs (**Figure 4A**). 500 targets are targeted in these studies, and are responsible for more than 500 diseases or clinical indications (**Figure 4A**).

IMGT/mAb-KG also contains mAbs with specific modifications to enhance their functional capacity or activation. We count more than 150 mAbs conjugated to drugs or molecules, we also have some mAbs radiolabeled with some radioactive substances and more than 175 mAbs fused with another protein or peptide (**Figure 4B**).

**Figure 5:**
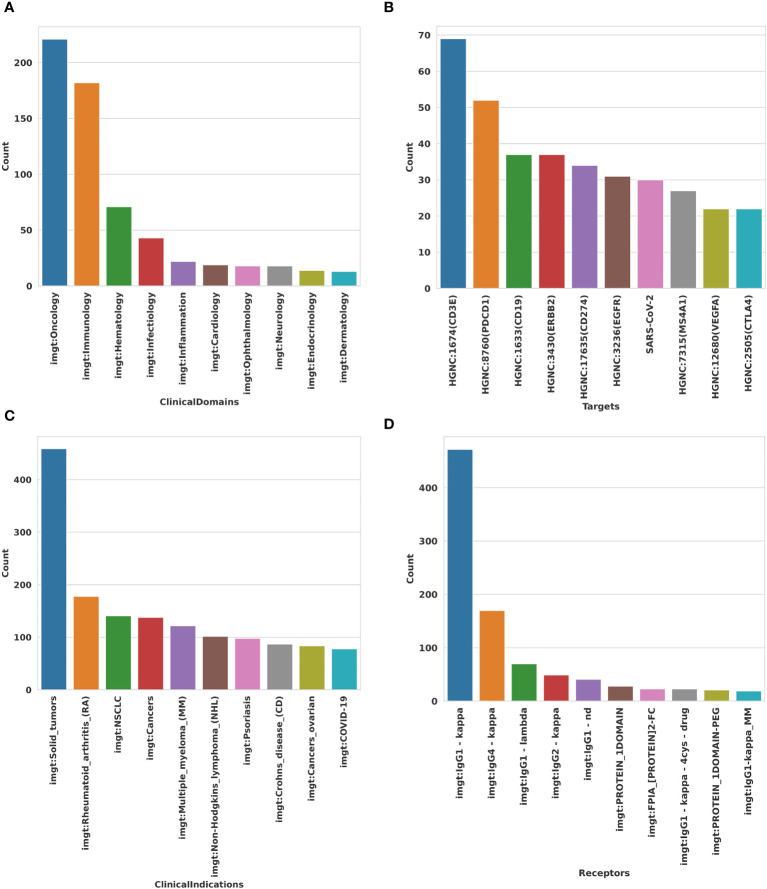
Representation of top 10 of some entities inside IMGT/mAb-KG. **(A)** Top 10 clinical domains where mAb targeted by mAb. **(B)** Top 10 molecules targeted by mAb are used. **(C)** Top 10 clinical indications (diseases) treated by mAb. **(D)** Top 10 receptors used in mAb engineered.

Typically, the majority of receptors utilized for engineered mAbs have a light chain Kappa, also known as antibody Kappa. This prevalence can be attributed to the fact that the initial mAbs were derived from mice, which naturally produce a high proportion (95%) of Kappa antibodies. However, there is now a growing presence of light chain Lambda in engineered mAbs, as mice are no longer the exclusive source of mAb production (**Figure 5D**).

In IMGT/mAb-KG, more than 475 mAbs are made with the IgG1-Kappa, an IG with a heavy chain Gamma (isotype 1) and a light Kappa chain (**Figure 5D**). The choice of IgG1-Kappa can be justified by not only its abundance, 66% in the human body, but also by its ability to activate the classic complement pathway, which is higher than the other IgG isotype (34): IgG1 ≻ IgG3 ≻ IgG2 and IgG4 are incapable of activating the complement. Therefore, IgG1 will be used when cytotoxic activity (antibody-dependent cell-mediated cytotoxicity (ADCC) and complement-dependent cytotoxicity (CDC)) is desired, as with mAb_1204 (tuvonralimab) (34). IgG2-Kappa or IgG4-Kappa will be preferred when neutralization is desired, as with mAb_1211 (nofazinlimab) (34, 35).

The most targeted molecule (in total by 69 mAb) is CD3E, the CD3 epsilon subunit of the T cell receptor complex (**Figure 5B**). This may be due to its central role in T Cell activation via the CD3 complex. Therefore, targeting CD3E can activate or suppress T cell activity. For example, they can redirect T cells to attack cancer cells or, conversely, suppress overactive T cell responses in autoimmune conditions (36, 37).

The mAbs inside IMGT/mAb-KG are used in multiple clinical indications correlated with the clinical domain(**Figures 5A, C**). More than 480 mAbs are used in solid tumors in the oncology domain. In the treatment of rheumatoid arthritis, over 190 mAbs are developed.

**Figure 6:**
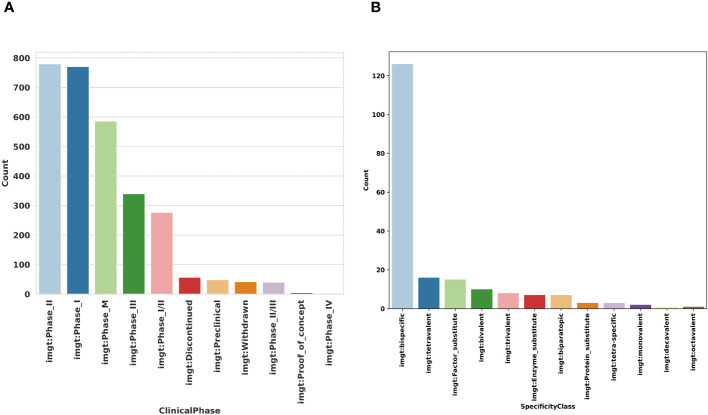
Representation of the Clinical phase and the specificity classes in IMGT/mAb-KG. **(A)** Clinical Phase in different clinical. **(B)** Specificity classes in mAb-DB trial (study products).

At this date, most of the clinical studies on the mAbs are in phase II or phase I (**Figure 6A**), approximately 600 mAbs are tested in different studies and approved for the commercialization. Besides the monospecific mAbs (targets one epitope of the antigen), we have over 120 bispecific mAbs (**Figure 6B**), considered as the next generation of mAbs due to their abilities to bind to two antigens or two epitopes of the same antigen simultaneously (38). By targeting two antigens or epitopes, they can cause multiple physiological or antitumor responses. These responses may be independent or connected (38).

### 3.2 IMGT/mAb-KG in use

IMGT resources are largely adopted by the international immunogenetics scientific community; it is the main reference in immunogenetics and immunoinformatics. IMGT/mAb-DB is used by clinicians, pharmaceutical industries and monoclonal antibody communities such as *Labex MAbImprove*. Thus, IMGT/mAb-KG targets the same users by providing free access and all the facilities of the knowledge graph-powered applications such as SPARQL endpoint for querying the graph. Then the user might have more flexibility to query information with an expressive query language. For example, a user might look for a specific mAb and its structural (proteins) information such as protein chains and domains information. For that, the user should construct a federated query, then combine information from IMGT/mAb-KG and IMGT-KG. So the user should select the mAb with its INN name (relatlimab for example) and the associated IMGT structural access number. The access number will be used as a bridge to IMGT-KG content, then the user should associate the protein crystal related to this access number, and the structures in the crystal and finally the chains associated to the structures. The user might select information about chains such as label, description, molecule component, amino acid sequences.

**Figure 7:**
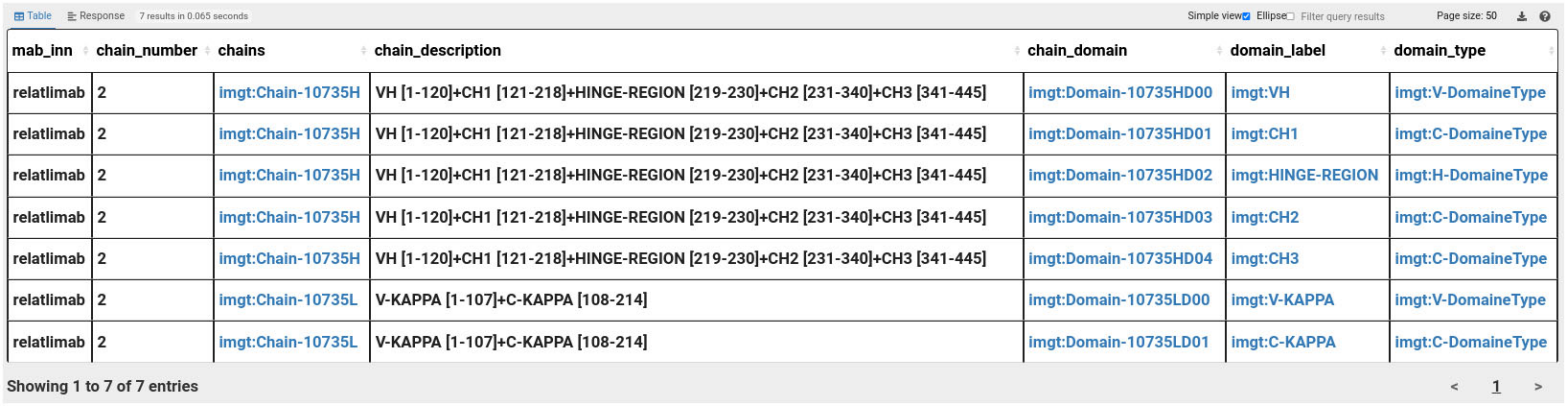
Integrative query results: relatlimab and the associated protein structure information.

**Figure 8:**
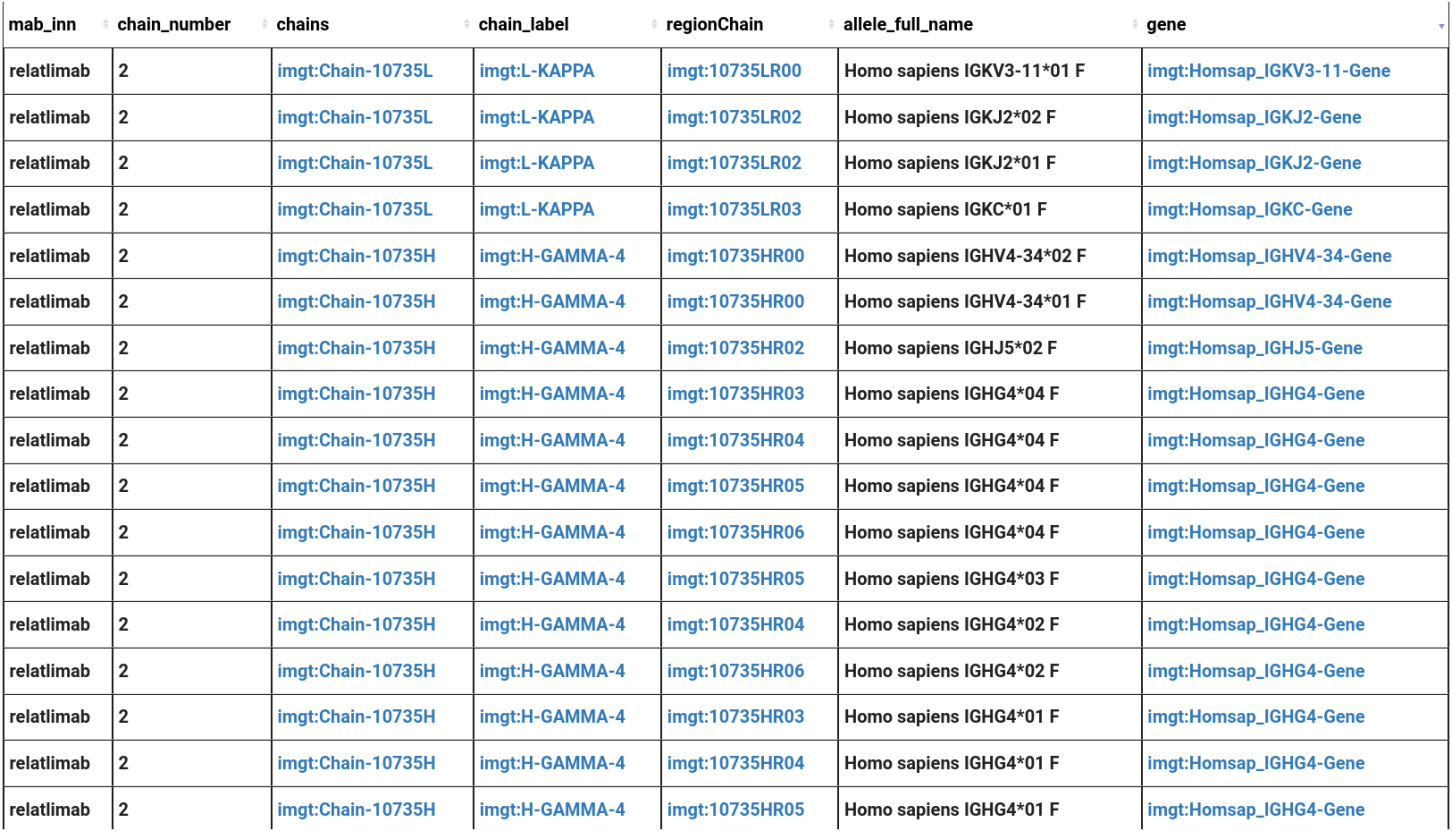
Integrative query results:relatlimab and the associated protein structure and genomics information.

From the results in **Figure 7**, we observe that the relatlimab is made up of two chains: Heavy Gamma 4 (H-GAMMA-4) and Light Kappa (L-KAPPA). In total, the relatlimab has seven domains: two domains for the L-KAPPA (V and C-KAPPA) and five for the H-KAPPA (VH, CH1 to CH3, and the Hinge Region). We also observe the description of each chain. The user might need to access to the associated genomics information such as gene or allele. To select the allele associated to these chains, the user should go through the different regions of the chains and the alignment associated to these regions, then each alignment is associated to an allele and alignment score, and the user can have access to the gene information via the allele (**Figure 8**).

**Figure 9:**
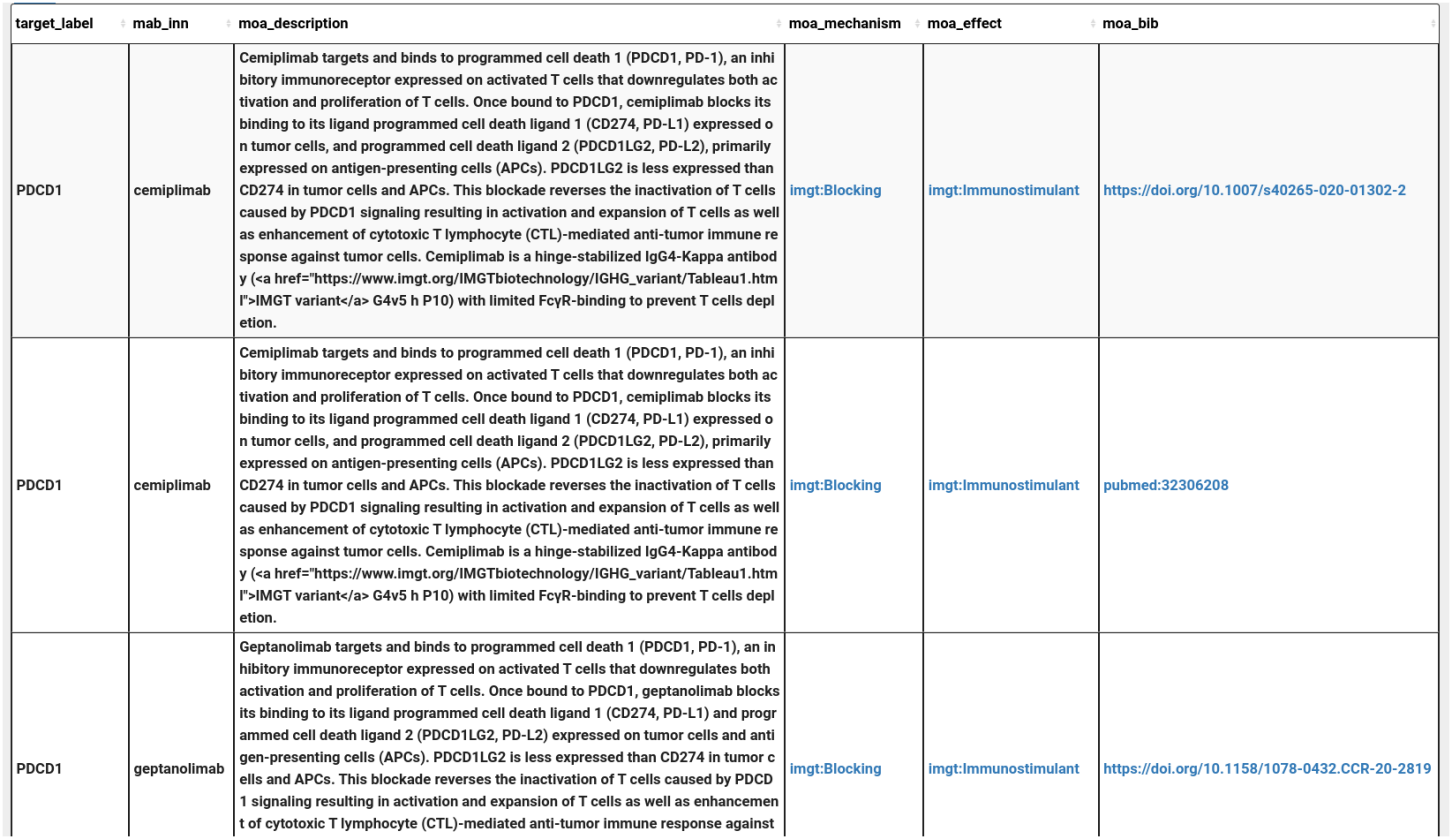
MOA query results (truncated): mAbs targeting PDCD1 with the description of their MOA.

The user might also need to search information (description, mechanism, effect, or bibliography) about the MOA of a given mAb (one or multiple) on a specific target for example (PDCD1: Programmed death receptor 1). For that, the user should select the mAb, then associate the related target, the MOA and its associated information such as description, mechanisms of action, the effect, the bibliographic references and the picture (**Figure 9**).

The flexibility of the SPARQL query interface allows the user to retrieve the mAbs’s paratope-epitope interactions and the contact analysis made by IMGT. To accomplish this, the user should select the mAbs of interest and then look at the IMGT structure entries associated. From these IMGT entries, the user should associated to the entries, the paratope-epitope interactions and the contact analysis links. The user can go further by looking at the interactions from IEDB, the immune epitope database and tools (39). All SPARQL queries are freely available on Zenodo.

**Figure 10:**
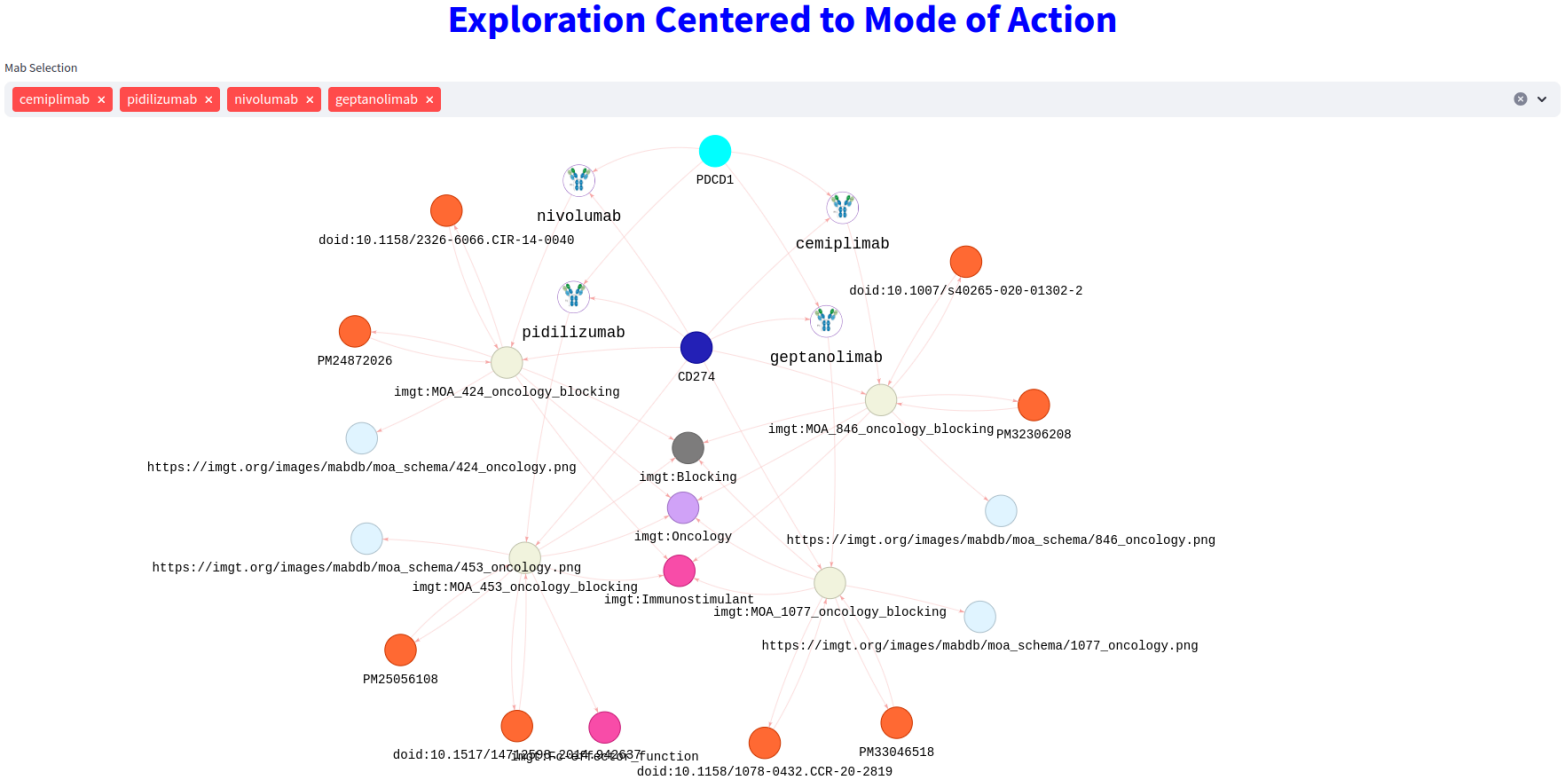
Exploration of MOA (truncated): mAbs targeting PDCD1 with the description of their MOA.

Considering the fact that users are not specialists in SPARQL queries, an adapted interface has also been implemented and freely available at https://www.imgt.org/mAb-KG/. The home page gives an overview of the content of the KG via various statistic charts. The IMGT/mAb-KG exploration page allows the user not only to navigate through different entities but also to view the graph of these entities, thus providing visual and easily adoptable support. This interface provides the user with the ability to customize queries with checkboxes at different levels. For example, in the **Figure 10**, the user can view the mAbs and their MOA (effect, bibliography, and picture) with the related target by simply typing the INN name of the mAbs. In addition, the user can select multiple mAb to see their interaction.

In addition to the exploration of the MOA, different other explorations centered on different types of entity are proposed for use, such as:

- Exploration centered on the mAb proposes to navigate in the different properties of the mAb including the target level, the construct level and the mode of action of mAb (if it exists). In this exploration, users can select different mAbs to see if they have similar properties (targets, disease)
- Exploration centered on the target gives information solely on the target.
- Exploration centered on the clinical indications or diseases shows different mAbs implied in the treatment of a given disease.
- Exploration centered on the product of the mAbs proposes to navigate between different mAbs products, including their study products and the decisions taken regarding these studies.

Additional information is given for each IMGT/mAbs node. When users double-click on IMGT nodes in the graph, they are redirected to the documentation for the node in question, providing additional information. In this way, additional information such as MOA descriptions are provided in such documentation. In addition, a redirection to the IMGT/mAb-DB database page is also made when the user double-clicks on a given mAb.

To make the IMGT/mAb-KG interface more readable, the users have the possibility to customize and restrict their searches (left-hand tab). For example, in the mAbs-centric exploration interface, the user has the option of showing or hiding information at different levels, such as mAbs, construction, target, or MOA level if it exists. This makes exploration and visualization smoother and easier to understand. Additionally, users can display the entire interface by hiding the left-hand tab, giving them a wider screen to explore.

Users can also turn on or off the printing of the relation name with the toggle button (PRINT_relation_name). A feedback form has been set up to receive user feedback and suggestions.

## 4 Discussion

These last years, we have witnessed the emergence of therapeutic mAbs in clinical indications including graft rejection, cancers, cardiovascular diseases, autoimmune diseases, etc. Showing good results on these indications (38), mAbs are considered today as a pivotal breakthrough in medical science, giving a hopeful horizon in the field of medical therapies.

To organize and provide access to the knowledge about the mAbs, IMGT has built IMGT/mAb-DB, a database for therapeutics mAbs. IMGT/mAb-DB provides access via a user-friendly web interface. However, this method limits personalized requests as well as isolates information from other databases.

We introduce IMGT/mAb-KG, a IMGT-KG (8) for monoclonal antibodies, integrating not only the data from IMGT/mAb-DB but also the related data of IMGT-KG. Then IMGT/mAb-KG allows the exploration of different omics levels of a mAb, including genomics and proteomics information. Counting in total 139,629 triplets, it provides access to 1489 mAbs, around 500 targets and over 500 clinical indications. IMGT/mAb-KG is linked to other resources such as Thera-SAbDab (32), pharmGKB, PubMed or HGNC making it an indispensable resource for the mAb development.

IMGT/mAb-KG will undergo active maintenance concurrently with updates to the IMGT databases and associated external resources. This will ensure that new versions of the graph will synchronize with those of IMGT databases like IMGT/mAb-DB, 3DStructure-DB, and 2D-Structure-DB. In forthcoming iterations, we aim to integrate it with the upcoming Patent and Literature Antibody Database PLAbDab (40). As for interface upkeep, it will adapt according to user requirements, with feedback being gathered to enhance its intuitiveness and accessibility.

In future work, we plan to develop a recommendation of a mAb system by applying representation learning models on IMGT/mAb-KG (41). In fact, for a given disease or target, we plan to propose to the users, the mAbs which are susceptible to target the given target or to treat the disease. This recommendation, boosted by the knowledge graph, aims to propose new potential mAbs candidates for existing targets and diseases, then accelerate mAbs development.

## Data availability statement

The datasets presented in this study can be found in online repositories. The names of the repository/repositories and accession number(s) can be found below: https://imgt.org/mAb-KG/, https://www.imgt.org/imgt-ontology, https://www.imgt.org/imgt-kg/mabkgdata.html, https://doi.org/10.5281/zenodo.11058211.

## Author contributions

GS: Conceptualization, Methodology, Software, Visualization, Writing – original draft, Writing – review & editing. TM: Data curation, Writing – review & editing. KT: Supervision, Writing – review & editing. VG: Supervision, Writing – review & editing. PD: Data curation, Resources, Software, Supervision, Visualization, Writing – review & editing. SK: Supervision, Writing – review & editing.
